# The curious incident of a cavum velum interpositum cyst in twins of a mother carrying May-Hegglin anomaly: a case report and short literature review

**DOI:** 10.1186/s12884-020-03437-2

**Published:** 2020-12-11

**Authors:** Giulio Giordano, Giovanni L. Tiscia, Giovanni Favuzzi, Elena Chinni, Mariano Intrieri, Mario Mastroianno, Letizia Di Meglio, Maurizio Margaglione, Elvira Grandone

**Affiliations:** 1 Hematology Clinic-Internal Medicine Dept. “A. Cardarelli” Hospital, Campobasso, Italy; 2 Thrombosis and Haemostasis Unit, Fondazione I.R.C.C.S. “Casa Sollievo della Sofferenza”, Viale Cappuccini, S. Giovanni Rotondo, 71013 Foggia, Italy; 3 Department of Medicine and Health Science “V. Tiberio”, University of Molise, Campobasso, Italy; 4 Scientific Direction, Fondazione I.R.C.C.S. “Casa Sollievo della Sofferenza”, S. Giovanni Rotondo, Foggia, Italy; 5 Department of Health Sciences, University of Milan, Milan, Italy; 6 Medical Genetics, University of Foggia, Foggia, Italy; 7 Ob/Gyn Department of The First I.M. Sechenov Moscow State Medical University, Moscow, Russia

**Keywords:** Pregnancy, May-Hegglin, Outcome, Case report

## Abstract

**Background:**

May-Hegglin anomaly is an autosomal dominant inherited condition, characterized by thrombocytopenia, giant platelets and Dohle-like bodies. Incidence is unknown and affected individuals can show from mild to moderate-severe haemorrhagic symptoms. The cyst of cavum veli interpositi (a virtual space filled with fluid within the third ventricle) is rarely reported in the foetal period. Furthermore, it is unclear whether isolated cavum veli interpositi cysts are a normal variant or developmental malformations. The simultaneous presence of these two anomalies was never described.

**Case presentation:**

We describe a very rare case of a twin monochorionic pregnancy in a woman with the May-Hegglin anomaly, whose foetuses carried cavum veli interpositi cysts. Since childhood, our patient had shown macro-thrombocytopenia, deafness and bleeding (epistaxis and menorrhagia), but she was misdiagnosed until the age of 30 years when our Centre identified a de novo allelic variant in the gene MYH9 coding for the non-muscle myosin heavy chain IIa. Our patient bled neither during the pregnancy, nor in the peripartum period. Children are now eight-months-old and have never bled, although both inherited the MYH9 variant and have thrombocytopenia with giant platelets. Furthermore, none of them developed psychomotor disorders.

**Conclusions:**

To the best of our knowledge, this is the sixth case of twin pregnancy in a woman carrying May-Hegglin anomaly and the first one with cavum veli interpositi cysts in the neonates. We speculate that MYH9 could have, at least in part, played a role in the development of both conditions, as this gene has a pleiotropic effect.

## Background

The May-Hegglin anomaly (MHA) is an autosomal dominant trait characterized by thrombocytopenia, giant platelets and Dohle-like bodies (in 42–84% cases, 15–100% of neutrophils) into cytoplasm neutrophil granulocytes [1]. It is caused by mutations in the gene MYH9 coding for the non-muscle myosin heavy chain IIa (NMMHC II-a), a cytoskeletal contractile protein.

Incidence of this anomaly is unknown and the clinical picture is quite heterogeneous, ranging from a mild to moderate bleeding tendency. Therefore, affected individuals can show epistaxis and bruising, as well as gum, heavy menstrual and postoperative bleeding. However, systematic reviews of pregnancies in MHA suggest that the course of pregnancy is not different from that of healthy women [2, 3]. In addition, the incidence of delivery-related and neonatal outcome bleedings is negligible [2].

Cavum veli interpositi (CVI) is a space within the double-layered telachoroidea of the third ventricle, that, when dilated and filled with fluid, results in the CVI cyst. It has been rarely reported in the foetal period (mean diameter 10–12 mm). Furthermore, it is still unclear whether isolated CVI cysts are normal variant or developmental malformations [4]. To date, foetuses with a cyst size > 7.1 mm are expected to undergo a more detailed brain examination [5].

We here describe a very rare case of a twin monochorionic pregnancy in a woman with MHA, whose foetuses carried CVI cyst. Since childhood our patient had shown macro-thrombocytopenia, deafness and bleeding tendency (epistaxis and menorrhagia), but she was misdiagnosed until 30 years when our Centre identified an MYH9 de novo allelic variant.

## Case presentation

**Fig. 1:**
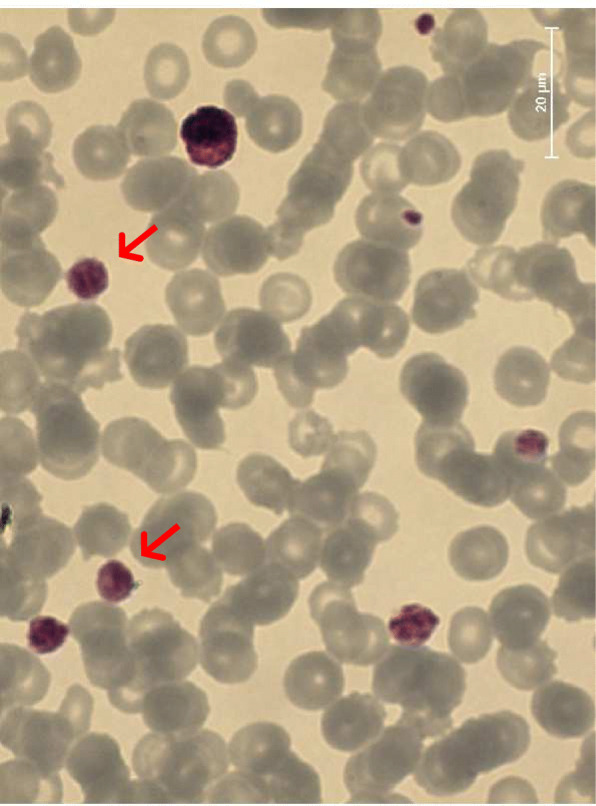
Blood smear image. Legend: Arrows point giant platelets

**Fig. 2:**
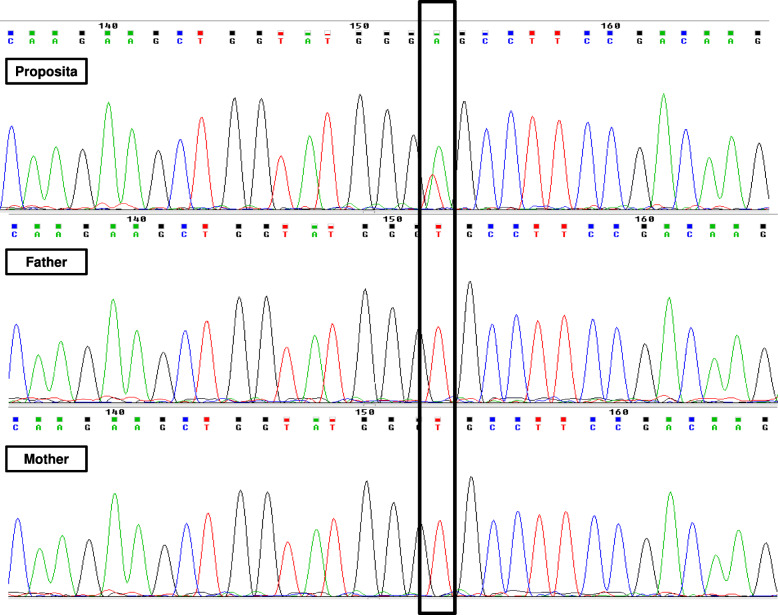
MYH9 Direct Sequencing. Legend: In the upper electropherogram, de novo c.101 T > A (p.Val34Glu) heterozygous variant in the proposita. Middle and lower electropherograms show wild-type MYH9 sequences in proposita’s parents

A 30-year-old woman came to our observation because of thrombocytopenia and mild haemorrhagic symptoms. Patient underwent laboratory investigations, which confirmed thrombocytopenia (platelet count = 9 × 10^9^/L) with high Red-Distribution-Width (RDW) value [RDW = 20.2% (11–16.5%)]. In addition, the blood smear examination showed giant platelets (Fig. 1). Light transmission aggregometry, performed on a whole blood sample, was within normal ranges. Presence of giant platelets and thrombocytopenia suggested molecular investigation for suspected MYH9 mutations. The patient carried a previously described de novo heterozygous transversion c.101 T > A (p.Val34Glu) in the MYH9 exon 2 (Fig. 2) that confirmed the diagnosis of MHA. Family members were also investigated: none showed macro-thrombocytopenia or bleeding episodes and, as expected, MYH9 mutations. She had been misdiagnosed with severe idiopathic thrombocytopenic purpura at the age of two yrs. Since childhood, she had suffered mild epistaxis and menorrhagia, that, however, were clinically irrelevant (she never received a diagnosis of anaemia). Notably, she also showed a documented diagnosis of mild sensorineural deafness, that further suggested the MYH9 diagnosis. She was treated with splenectomy at the age of 15 years and chronic discontinuous prednisone administration (25 mg/day) for a short time, when platelet count fell below 30 × 10^9^/L.

**Fig. 3:**
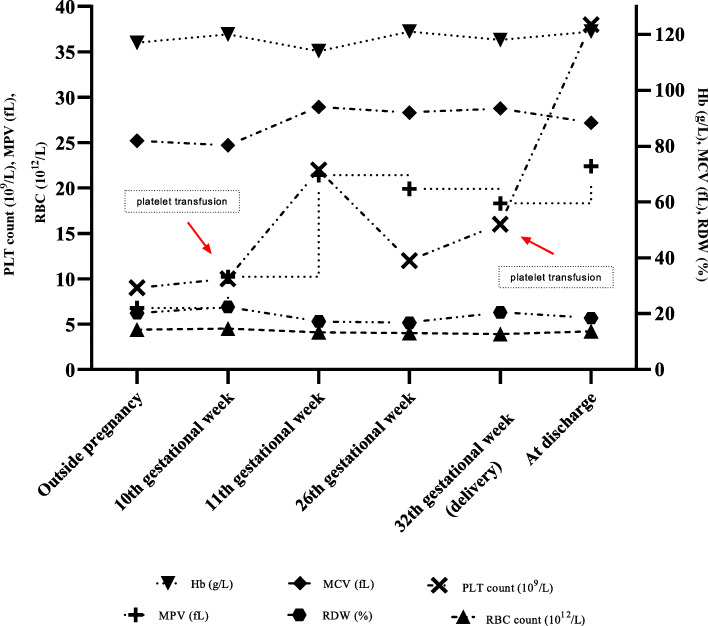
Variation of blood parameters outside and throughout pregnancy. Legend: Point and connecting line graph show fluctuation of blood parameters in the proposita. Hb (Hemoglobin), MCV (Mean Corpuscular Volume), PLT (Platelet), MPV (Mean Platelet Volume), RDW (Red blood cells Distribution Width), RBC (Red Blood Cell).

**Fig. 4:**
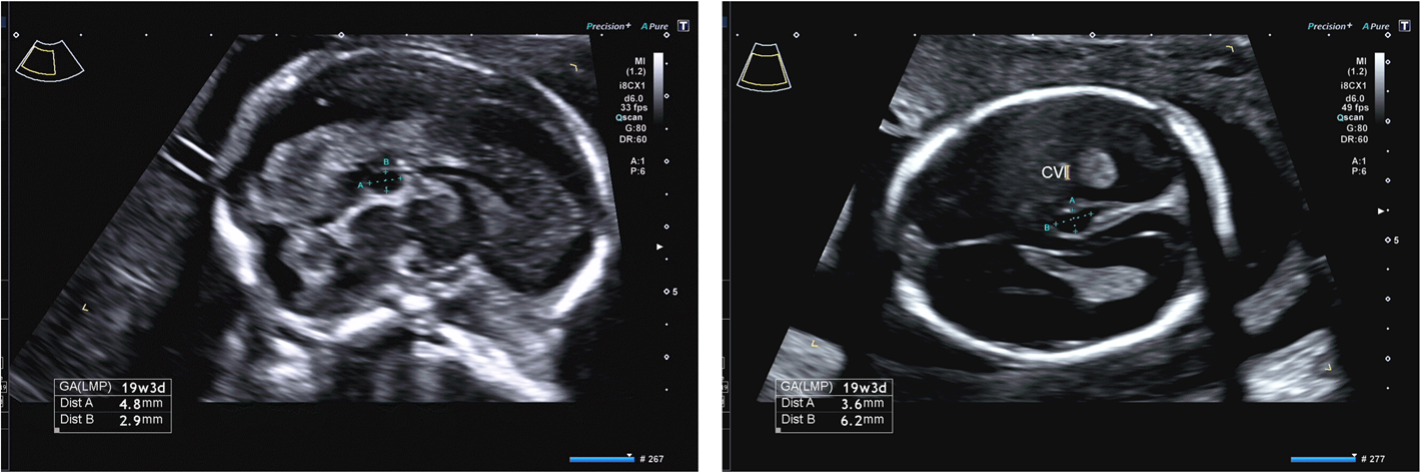
Ultrasound images  of the cyst in both foetuses. Legend: Sagittal view of the cyst in the foetus **a** (left), axial view of the cyst in the foetus **b** (right). The cyst size can be seen in the bottom left corner of both images

In 2016, she came to our observation and was diagnosed with MHA. In 2018 she got pregnant: a monochorionic biamniotic pregnancy was diagnosed. The pregnancy was carefully monitored on both maternal and foetal side. Variations in blood parameters measured throughout pregnancy are shown in Fig. 3. The patient received two units of platelets also at the 10th week of gestation, because platelet count fell down below 10 × 10^9^/L, although she never showed bleeding symptoms or anaemia. At the 19th week, foetal ultrasound demonstrated in both twins “isolated small CVI cyst” (4.8 × 2.9 mm and 6.2 × 3.6 mm, respectively) (Fig. 4). At this time foetal biometry and anatomy, placental insertion and morphology, Doppler velocimetry and hemodynamic imaging were all normal; signs of twin -to -twin transfusion syndrome were absent. At the 32nd week of gestation, she delivered by means of a caesarean section, because anomaly in the Doppler velocimetry was observed and foetal growth restriction in one of the twins was diagnosed. Before delivery, she had platelet count of 16 × 10^9^/L and was therefore transfused with two units of platelets. Furthermore, she received a single dose of intravenous tranexamic acid 1 g before general anaesthesia and gave birth to two males (birth weight: 1500 and 2100 g, respectively), who both showed thrombocytopenia and giant platelets. Both were in good clinical conditions and, as expected, one of them was below the 10th percentile. Magnetic Resonance confirmed the diagnosis and the size of the CVI cysts dimension in both newborns. Genetic analysis evidenced in both the presence of the heterozygous transversion c.101 T > A (p.Val34Glu) in the MYH9 exon 2. Neither mother nor newborns experienced bleeding symptoms. After 6 days, the mother was discharged with a platelet count of 38 × 10^9^/L, while twins were admitted to Neonatal Intensive Care Unit; they were discharged after 28 days. Twins are closely followed by the paediatrician and undergo clinical and laboratory follow-up. Both are in good health, without bleeding or psychomotor disorders. The last blood count, performed at eight months of age, showed platelet count of 33 × 10^9^/L and 53 × 10^9^/L, respectively.

## Discussion

We describe a very rare case of monochorionic biamniotic pregnancy in a patient with MYH9-related disease (MYH9-RD); the presence of the CVI cysts in both twins confers on this case a unique character. The first relevant issue to underline is the absence of bleeding episodes, as well as other maternal complications, even in the presence of an obstetric condition as monochorionic diamniotic pregnancy, that is often associated with complications [6]. This reflects the mild phenotype associated with the mutation carried by our patient. She was only prudentially transfused at the 10th week, because her platelet count fell below 10 × 10^9^/L. Furthermore, she received two additional platelet units before delivery as haemostatic prophylaxis.

Two systematic reviews of pregnancies carried out by women with MHA suggest that the course of pregnancy in these patients does not differ from that of healthy women. On the other hand, the risk of bleeding during childbirth seems higher in both mothers and neonates [2]. However, delivery-related and neonatal bleedings are quite low (six out 94 newborns), although occasional fatal haemorrhages have been described (two neonates, both born by vaginal delivery to mothers with severe thrombocytopenia).

In the comprehensive systematic review carried out by Hussein et al., it has been calculated that postpartum haemorrhage (PPH) has 5% rate among the women with MHA [3], suggesting a low prevalence of bleeding manifestations in this setting. PPH was documented in two out of the 59 (3.4%) pregnancies described: in both cases, a haemostatic prophylaxis in the peripartum period was not performed [7, 8]. Conversely, PPH was also described in women with haemostatic prophylaxis prior to delivery [7, 9, 10]. In agreement with these findings, we found that pregnancy did not confer risk for bleeding manifestations.

CVI cysts are more common in foetuses with brain anomaly, but isolated cysts of CVI are usually associated with favourable postnatal outcome. Indeed, it has been reported that prenatal vs. postnatal diagnoses of CVI cysts seem to be associated with a normal child development [4]. A recent systematic review reports on 47 foetuses carrying CVI cysts and none of those with isolated cysts had an adverse outcome [11]. Clinical significance of CVI cysts in the newborns described here is consistent with the available literature in the field: indeed, until now twins show a healthy neurological development. However, they deserve a long-term follow-up with a special focus on neurological development.

**Fig. 5:**
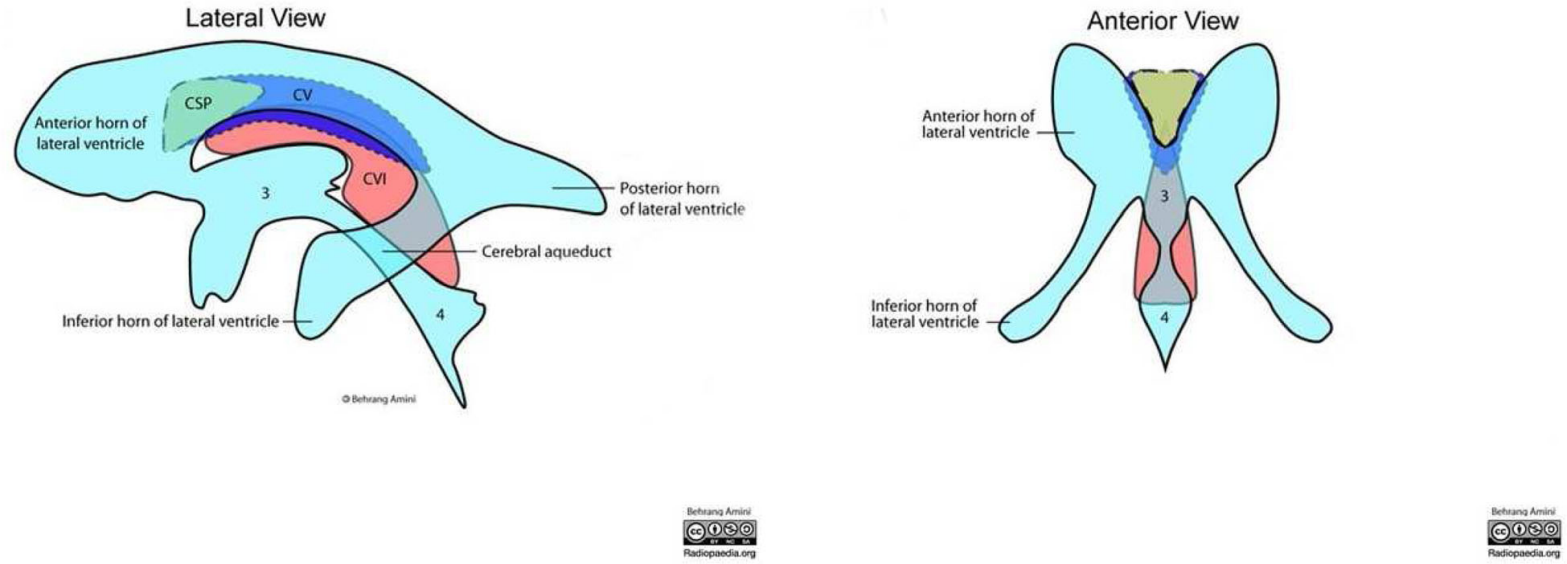
Brain anatomical site of the CVI cysts. Legend: Diagram illustrates lateral and anterior views of the brain anatomical site affected by the CVI cysts. CSP: Cavum Septum Pellucidum; CV: Cavum Vergae; CVI: Cavum Velum Interpositi; 3 = 3rd ventricle; 4 = 4th ventricle

To the best of our knowledge, this is the sixth case of twin pregnancy [3] in a woman carrying MHA and the first one with CVI cysts in the neonates. At variance with previous reports, we observed the association of two rare conditions: the CVI cyst and the MHA [4]. The site of CVI cyst is the forebrain (Fig. 5), where the expression of the MYH9 has been shown to have a central role in morphogenesis, and in particular in the cell shape changes. Furthermore, in zebrafish embryo, MYH9 regulates the cell shortening during the brain morphogenesis [12]. Therefore, it is conceivable that mutations within MYH9 might play a role in the impaired brain development. We hypothesise that in these twins, the cysts development might have been influenced by the MYH9 gene. However, more research is needed to investigate whether MHA and CVI cysts are cards of the same puzzle or this association is just “a curious incident”. The coexistence of prematurity, FGR and MHA could have facilitated intracerebral bleeding in these twins. The uneventful delivery outcome and the clinical and laboratory follow-up confirm the relatively low bleeding risk associated with MHA.

## Conclusions

Notwithstanding the limitation of findings from a case report article, the case reported here underlines how challenging is the management of MHA in pregnancy and how joint cooperation between obstetrician and haematologist is crucial to minimize foetal and maternal risks.

## Data Availability

The datasets used and/or analyzed during the current study are available from the corresponding author on reasonable request.

## Abbreviations

CVI: Cavum veli interpositi
MHA: May-Hegglin anomaly
MYH9: Myosin-9
MYH9-RD: MYH9-related disease
NMMHC II-a: Non-muscle myosin heavy chain IIa
PPH: Postpartum haemorrhage
RDW: Red-Distribution-Width
